# Human ESC‐derived immunity‐ and matrix‐ regulatory cells ameliorated white matter damage and vascular cognitive impairment in rats subjected to chronic cerebral hypoperfusion

**DOI:** 10.1111/cpr.13223

**Published:** 2022-04-19

**Authors:** Yilong Zhao, Jun Wu, Da Li, Jing Liu, Weiqi Chen, Zongren Hou, Kailun Liu, Lingling Jiang, Xiaowei Chen, Liu Wang, Baoyang Hu, Fangrong Zong, Yukai Wang, Yilong Wang

**Affiliations:** ^1^ Department of Neurology, Beijing Tiantan Hospital Capital Medical University Beijing China; ^2^ China National Clinical Research Center for Neurological Diseases Beijing China; ^3^ Advanced Innovation Center for Human Brain Protection Capital Medical University Beijing China; ^4^ Beijing Key Laboratory of Translational Medicine for Cerebrovascular Disease Beijing China; ^5^ State Key Laboratory of Stem Cell and Reproductive Biology, Institute of Zoology Chinese Academy of Sciences Beijing China; ^6^ Institute for Stem Cell and Regeneration Chinese Academy of Sciences Beijing China; ^7^ Beijing Institute for Stem Cell and Regenerative Medicine Beijing China; ^8^ National Stem Cell Resource Center Chinese Academy of Sciences Beijing China; ^9^ University of Chinese Academy of Sciences Beijing China; ^10^ Savaid Medical School University of Chinese Academy of Sciences Beijing China; ^11^ Institute of Biophysics Chinese Academy of Sciences Beijing China; ^12^ School of Artificial Intelligence Beijing University of Posts and Telecommunications Beijing China; ^13^ Chinese Institute for Brain Research Beijing China

## Abstract

**Objectives:**

This study investigated the ability of immunity‐ and matrix‐ regulatory cells (IMRCs) to improve cognitive function in a rat model of vascular cognitive impairment.

**Materials and Methods:**

A chronic cerebral hypoperfusion (CCH) model was established in rats via permanent bilateral occlusion of the common carotid arteries (two‐vessel occlusion, 2VO). The rats then received intravenous injections of IMRCs or saline. A single injection of different doses of IMRCs (1 × 10^6^ cells/rat, 2 × 10^6^ cells/rat, or 4 × 10^6^ cells/rat) was administered via tail vein 72 h after establishment of the model. To evaluate functional recovery, the rats were subjected to behavioural tests after 30 days of CCH. Imaging, western blotting, immunofluorescence staining, and quantitative real‐time PCR were used to analyse neuroinflammation and white matter injury after 14 and 40 days of CCH. RNA sequencing (RNA‐seq) was used to profile gene expression changes in copine 1 (CPNE1) in response to IMRCs treatment.

**Results:**

Intravenous injection of 4 × 10^6^ IMRCs alleviated white matter damage and ameliorated cognitive deficits in rats subjected to CCH. Immunofluorescence staining suggested that activation of microglia and astrocytes was reduced, and RNA sequencing showed that CPNE1 expression was significantly elevated following treatment with IMRCs.

**Conclusions:**

Intravenous injection of IMRCs protected against CCH‐induced white matter injury and cognitive impairment inhibition of microglial activation and regulation of microglia polarization.

## Graphical abstract

Diagram of proposed action of IMRCs on vascular cognitive impairment. Intravenous injection of IMRCs protected against CCH‐induced white matter injury and cognitive impairment through polarization of microglia and astrocytes.

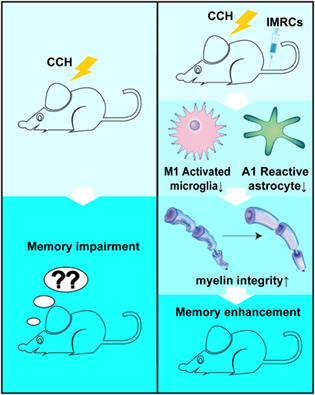

## 1 INTRODUCTION

Vascular cognitive impairment (VCI) includes a range of impairments from subjective cognitive decline to dementia, and can be caused by the entire spectrum of vascular brain pathologies. Vascular cognitive impairment is the second leading cause of dementia, following Alzheimer's disease (AD).^1^ The main symptoms of VCI include mental slowness, and issues with memory and executive function. Other neurological signs, such as behavioural symptoms and psychological symptoms, occur frequently.^2^ Activation of microglia and astrocytes, accompanied by oligodendroglia apoptosis, is the major biochemical change in VCI pathophysiological.^3^ No drugs have been approved for treatment of VCI.^4^ Therefore, there is an unmet need for new treatments to improve cognitive impairment resulting from VCI.

Chronic cerebral hypoperfusion (CCH) is the main cause of vascular cognitive impairment and dementia,^5^ and may result in blood–brain barrier (BBB) dysfunction, leading to increased extravasation of plasma proteins into the brain, which subsequently triggers an excessive inflammatory response.^6^ A CCH rat model has been widely used to investigate CCH.^7^ In this model, there is a decline in cerebral blood flow (CBF) in the cerebral cortex and corpus callosum. Hypoxia‐ischemia induces activation of multiple inflammatory pathways in microglia and astrocytes, such as the NF‐κB signalling pathway, ultimately leading to white matter injury and VCI.^8^, ^9^ In addition, white matter damage has been shown to be an important cause of cognitive deficits in CCH model.^10^ Copine 1 (CPNE1) is a Ca^2+^‐dependent phospholipid binding protein that contains a calcium‐dependent phospholipid binding domain (C2 domain, N‐terminal) and plasma and extracellular matrix protein binding domains (A domain, C‐terminal).^11^ Previous reports showed that CPNE1 was involved in TNF‐α‐induced suppression of NF‐κB transcriptional activity.^12^, ^13^

Stem cell therapy has provided new hope for treatment of central nervous system (CNS) diseases.^14^, ^15^, ^16^ In particular, mesenchymal stem cells (MSCs) have emerged as promising stem cell therapies due to their immune regulatory potential.^17^, ^18^ However, very few studies have evaluated the efficacy of MSCs for treatment of CCH induced by the 2VO approach.^19^ Moreover, donor heterogeneity of primary MSCs is a major hurdle to clinical use.^20^ Previously, we generated an hESC‐derived MSC‐like cell population which has unique abilities to modulate immunity and regulate extracellular matrix production. Therefore, we named these cells immunity‐ and matrix‐regulatory cells (IMRCs).^21^ Our previous studies suggested that IMRCs exerted therapeutic effects mainly through regulation of immune function in mouse models of AD and lung injury.^21^, ^22^

In this study, we investigated whether transplantation of IMRCs could improve VCI in a CCH rat model. Our findings suggested that IMRCs administered via tail‐vein injection suppressed activation of microglia and astrocytes, reduced white matter damage, and improved memory capacity and cognitive deficits in rats subjected to CCH. Using transcriptome sequencing, we demonstrated that CPNE1 expression was markedly increased following treatment with IMRCs. This finding suggests that IMRCs may represent a unique approach to treatment of VCI.

## 2 MATERIALS AND METHODS

### 2.1 Animals and subgrouping

All animal experiments were approved by the Animal Care and Use Committee of the Institute of Zoology, Chinese Academy of Sciences. Male SD rats of 7–8 weeks of age, weighing 280–320 g, were purchased from Vital River Laboratory Animal Technology Co., Ltd. The rats were housed at the Laboratory Animal Center of the Institute of Zoology with free access to water and lab chow and were maintained under a 12 h light/dark cycle. The rats were randomly divided into the following groups: (1) 2VO: both common carotid arteries (CCAs) were occluded permanently using nylon filament; (2) Sham: same surgical procedure as 2VO group but neither of the CCAs were occluded; (3) IMRCs: Three doses of IMRCs (1 × 10^6^, 2 × 10^6^, or 4 × 10^6^ suspended in 200 μl of 0.9% NaCl) were administered by intravenous injection 72 h after CCH. Rats in the sham and 2VO groups received 200 μl of 0.9% NaCl alone. The experimental schedule is illustrated in Figure 2A.

### 2.2 Rat model of CCH and cerebral blood flow measurement

Cerebral blood flow was measured using laser Doppler flowmetry. After being anaesthetised with 5% chloral hydrate, the rats were placed in a supine position on a heating pad at 37°C. A midline scalp incision exposed the skull. Using an electric drill, a burr hole to fix the tip of the LDF probe was made over the right parietal cortex (3 mm caudal from bregma and 5 mm lateral from the sagittal suture). The CCH model was established using the 2VO method as described previously.^23^ Cerebral blood flow values were expressed as a percentage relative to the baseline value. Successful CCH modelling was defined as a decrease in CBF to 30%‐ 40% of baseline.^24^

### 2.3 Morris water maze test (MWM)

The Morris water maze is a circular water tank divided into four quadrants. The water temperature was maintained at 24 ± 2°C. The escape platform (10 cm in diameter) was fixed in the center of the south‐east quadrant (target quadrant) and submerged about 1 cm below the water surface. During training, rats were gently placed into the water maze and released facing the wall from one of four quadrants in a random order, then allowed to swim freely to find the hidden platform. The rats were given 90 s to locate the hidden platform, and the latency to reach the escape platform was recorded. If a rat failed to find the platform within 90 s, it was guided to the platform and allowed to stay there for 15 s. Each rat was tested four times per day starting from different quadrants, with intervals of 20 min between attempts, and the average was used as the daily score. The probe trial was performed 24 h after the last training trial. Each rat was allowed to swim freely in the tank with the platform removed for 90 s. The time spent in target quadrant, the number of times the rat crossed the original platform and swimming speed were automatically measured using video tracking software (EthoVision, Noldus, Netherlands).

### 2.4 Barnes maze test (BMT)

The Barnes maze test was used to evaluate spatial learning and memory in each group at 30 days after CCH. The apparatus consists of a rotatable circular platform (1.22 m in diameter and 1 m from the floor) with 16 holes in the periphery. A hidden black escape box (20 × 15 × 12 cm) was located under one of the holes. A curtain with visual cues surrounded the maze pool to allow the rats to learn the position of the target hole. Maze testing was performed as described previously with minor alterations.^25^ Briefly, the test included three parts: habituation, acquisition training, and acquisition probe. During the habituation phase, the rats were placed into the escape chamber for 1 min, then left free to explore the platform for 3 min. The rats were then gently guided toward the escape chamber. The acquisition phase consisted of 5 consecutive training days with 2 trials per day. On each trial, the rats were placed in the start box resting on the surface of the platform. After an interval of 5–10 s the box was raised, and the rats were allowed to explore the maze. All rats were given 4 min to locate the escape hole, after which they were guided to the escape hole and placed in the escape box for 1 min. Maze was cleaned with 70% ethanol between trials to eliminate any potential odour cues. For each trial, primary errors and escape latency were measured using the software (EthoVision). Primary errors were calculated as the number of head deflections or pokes into incorrect holes before reaching the target hole. Escape latency was defined as the time spent before entering the escape box. The probe trial was carried out 24 h after the last training event. On the probe test day, the escape box was removed, and the time spent in the quadrant where the escape box was originally located was recorded during a 4 min period.

### 2.5 Novel object recognition (NOR) test

The novel object recognition test is commonly used in rodents to measure non‐spatial memory. The test was performed in a 72 × 72 × 35 cm open‐field box with objects located opposite the starting point. The day before testing, rats were allowed to freely explore the open arena for 5 min. On the testing day, the animals were placed in the familiar arena with two identical objects added and left to explore for 5 min. After a 24 h interval, the rats were put back in the same arena to explore one familiar object (cylinder) and one novel object (cuboid) for 5 min. The time that the animals spent exploring familiar objects (F) and novel objects (N) was recorded. The discrimination index (DI) was calculated as N/(N + F) × 100%.

### 2.6 Diffusion tensor imaging (DTI)

Magnetic resonance imaging (MRI) data of the rats were obtained at 1 month after surgery. They were initially anaesthetised by inhalation using a mixture of oxygen and 3%–4% isoflurane and then maintained using 0.8%–1.2% isoflurane. An MR‐compatible small‐animal monitoring system (SA Instruments Inc) was used to monitor the respiration rate and rectal temperature continuously throughout the entire experiment.

Under anaesthesia with isoflurane, the rats were placed in a prone position in an 11.7 Tesla pre‐clinical MRI scanner (Bruker) equipped with a rat head coil. DTI data were acquired using the following parameters: repetition time (TR) = 8500 ms; echo time (TE) = 17.5 ms; slice thickness = 0.35 mm; field‐of‐view (FOV) = 2.1 × 1.5 cm^2^; matrix = 110 × 82; number of averages (NA) = 2. The diffusion‐weighted images were sampled using the b value of 1000 s/mm^2^ (b1000) along with 35 non‐collinear, uniformly distributed directions gradient directions, and two images with b value of 0 s/mm^2^ (b0). T2‐weighed imaging (T2WI) was performed using a fast‐spin echo sequence with the following parameters: TR = 3500 ms; TE = 10 ms; slice thickness = 0.6 mm; FOV = 3.5 × 3 cm^2^; matrix = 256 × 220; NA = 2.

Raw DTI data were transferred into NIFTI format using DSI Studio and processed using FSL Software v6.0. The data were motion‐corrected using the EDDY_CORRECT function with the b0 volume as a reference, skull‐stripped using the RATS protocol (https://www.iibi.uniwa.edu/rats-rodent-brain-mri) and then refined and modified manually. The N4 bias field correction in the Advanced Normalization Tools was performed to correct for field inhomogeneity. The FSL Diffusion Toolkit (DTIFIT) was used for local fitting of diffusion tensors and to generate maps of fractional anisotropy (FA) and radial diffusivity (RD). DTI parametric maps were registered to the SIGMA Wistar rat brain template after registering the b0 images to T2 images.^26^

The corpus callosum, where white matter tracts are the most abundant in the rat brain, were selected as ROIs. These ROIs were extracted automatically based on the SIGMA Wistar rat brain atlas and transferred to identical sites on other parameter maps using the inversed registering transformation matrix. The representative values of FA and RD were then calculated in these ROIs using the FSLSTATS function.

### 2.7 Immunofluorescence staining

Rats were anaesthetized with 5% chloral hydrate (400 mg/kg body weight) and transcardially perfused with cold PBS followed by 4% paraformaldehyde (PFA). The brains were removed, post‐fixed in 4% PFA overnight at 4°C, then dehydrated in 30% sucrose. Then, the brains were coronally sliced into 35‐μm sections using a cryostat (Leica SM2010 R) and stored at −20°C in cryoprotective storage solution (125 ml of ethylene glycol, 125 ml of glycerol, and 150 ml of 0.1 M phosphate buffer). For immunohistochemical staining, the sections were washed with PBS three times, blocked with 5% BSA and 1% Triton X‐100 in PBS at room temperature for 2 h, then incubated with primary antibodies (diluted in 1% BSA, 0.2% Triton X‐100 in PBS) overnight at 4°C. The following primary antibodies were used: rabbit monoclonal anti‐MBP (1:1000; ab218011; Abcam), rabbit polyclonal anti‐Olig2 (1:1000; AB9610; Millipore), mouse monoclonal anti‐GFAP (1:300; MAB360; Millipore), rabbit monoclonal anti‐C3 (1:300; AB200999; Abcam), rabbit polyclonal anti‐Iba1 (1:200; 019–19,741; Wako), mouse monoclonal anti‐CD68 (1:100; MCA341; Bio‐Rad), mouse monoclonal anti‐HNA (1:200; ab191181; Abcam), mouse anti‐STEM121 (1:500; Y40410; TAKARA). After washing, the sections were incubated with AlexaFluor fluorophore (Alexa Fluor 488, 594 and 647)‐coupled secondary antibodies. Finally, the sections were sealed with mounting medium containing DAPI (ZLI‐9557; Zhongshanjinqiao, Beijing, China).

### 2.8 Western blotting

Brain tissue was dissected quickly from the corpus callosum after deep anaesthesia. The samples were processed for western blotting analysis as previously described.^22^ The primary antibodies used were: rabbit monoclonal anti‐MBP (1:1000; ab218011; Abcam), rabbit polyclonal anti‐Olig2 (1:1000; AB9610; Millipore), and mouse monoclonal anti‐β‐actin (1:4000; 30101ES60; Yeasen). After incubation with horseradish peroxidase (HRP)‐conjugated goat anti‐rabbit or goat anti‐mouse secondary antibody (1:5000; ZB5301/SA00001‐1; Zhongshanjinqiao/Proteintech), and bands were detected using enhanced chemiluminescence reagent (ECL, Pierce) and quantified using ImageJ software.

### 2.9 Quantitative RT‐PCR

Brain tissue was rapidly removed from the corpus callosum and the mRNA levels of M1 macrophage markers (CD86, iNOS) and M2 macrophage markers (CD206, Arg1), and inflammatory factors (IL‐1β, TNFα, IL‐6, IL‐10), were measured using quantitative real‐time PCR (qRT‐PCR). Total RNA was extracted using TRIzol reagent and complementary DNA synthesis was performed using a Revert Aid First Strand cDNA Synthesis Kit (Yeasen) according to the manufacturer's instructions. Quantitative real‐time PCR was performed using a SYBR Green Real Time PCR Master Mix (Yeasen) on a StepOne Plus Real‐Time PCR System (Applied Biosystems). Beta actin was used as the reference genes. All quantitative PCR was performed in triplicate using four independent purified RNA samples. The primer sequences are listed in Table 1.

**TABLE 1:** Primers used for qRT‐PCR

Gene	Primer sequences (5′–3′)	
CD86	F: CTCATCTAAGCAAGGATACCCGAAACC	R: TGGAAGAGATAGGCTGATGGAGACAC
iNOS	F: TCTTGGAGCGAGTTGTGGATTGTTC	R: AGTGATGTCCAGGAAGTAGGTGAGG
CD206	F: ACTGCGTGGTGATGAAAGG	R: TAACCCAGTGGTTGCTCACA
Arg‐1	F: AGAGGAGGTGACTCGTACTGTGAAC	R:TCTGGCTTATGATTACCTTCCCGTTTC
IL‐1β	F: AATCTCACAGCAGCATCTCGACAAG	R: TCCACGGGCAAGACATAGGTAGC
IL‐6	F: AGTTGCCTTCTTGGGACTGATGTTG	R: GGTATCCTCTGTGAAGTCTCCTCTCC
TNF‐α	F: ATGGGCTCCCTCTCATCAGTTCC	R: CCTCCGCTTGGTGGTTTGCTAC
IL‐10	F: GGCAGTGGAGCAGGTGAAGAATG	R: TGTCACGTAGGCTTCTATGCAGTTG
β‐Actin	F: GCCACCAGTTCGCCATGGAT	R: CATCACACCCTGGTGCCTAG

### 2.10 RNA‐seq analysis

Library preparation and sequencing was performed by BGI. Sequencing libraries were generated using the NEBNext UltraTM RNA Library Prep Kit for Illumina (NEB) according to the manufacturer's instructions. After cluster generation, the library preparations were then sequenced on an Illumina platform and 125 bp‐150 bp paired‐end reads were generated. Differential expression between groups was analysed using the DESeq2 package in R (1.16.1), with the filtering threshold set at an absolute fold change (FC) > 2 and the corrected p value <0.05. Enrichment of DEGs and gene ontology (GO) enrichment analysis were performed using the cluster Profiler package in R.

### 2.11 Data expression and statistical analysis

Analysis was performed using GraphPad Prism (Version 7.00, GraphPad Software Inc.). All data were presented as the mean ± SEM. Comparisons between the groups were statistically evaluated using Student's *t*‐test or one‐way analysis of variance (ANOVA) with a post hoc Tukey test. *p*‐values <0.05 were considered to be statistically significant.

## 3 RESULTS

### 3.1 Rats subjected to CCH exhibited decreased CBF and impaired cognitive function

**FIGURE 1:**
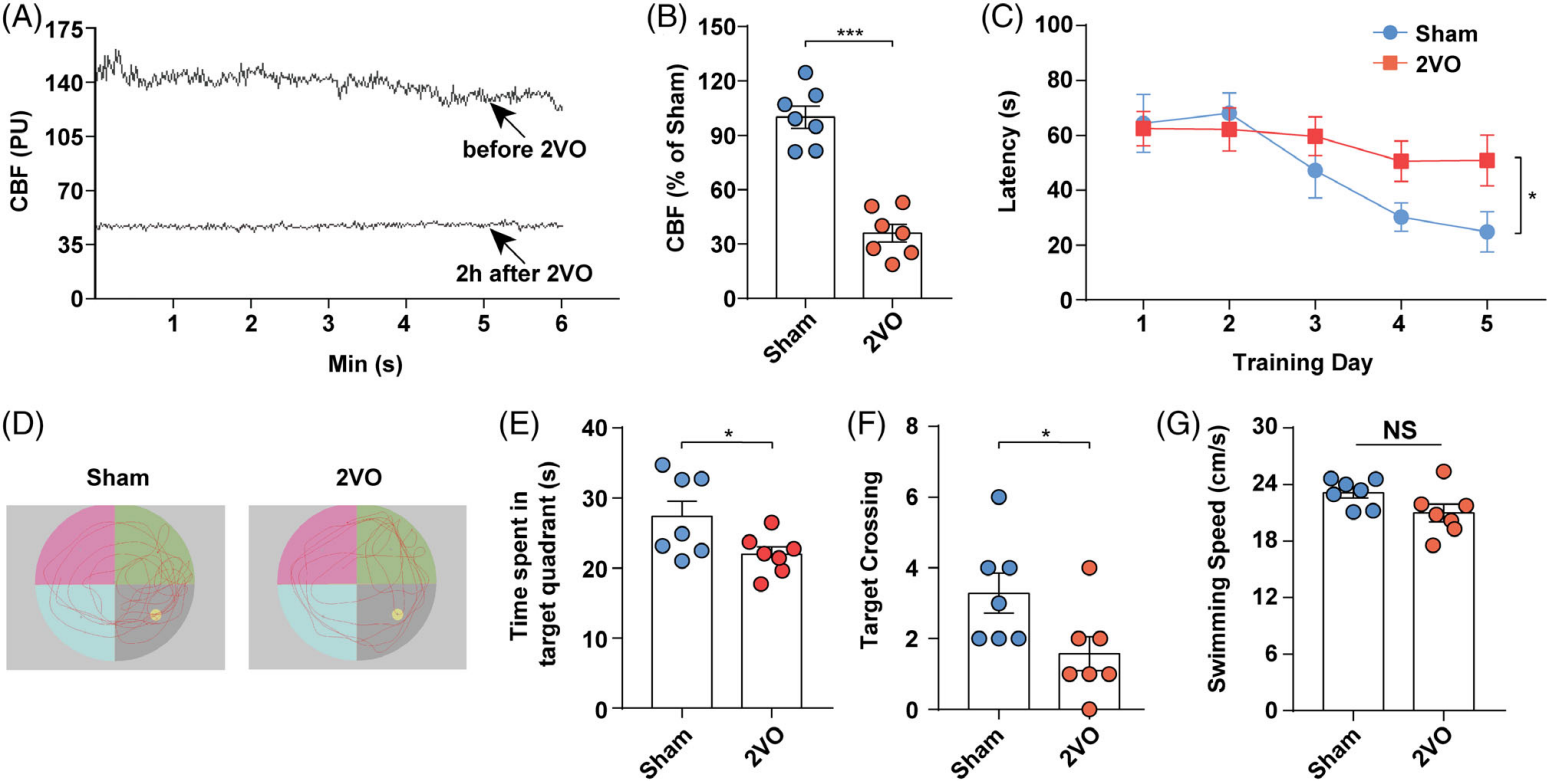
Rats subjected to CCH exhibited decreased CBF and impaired cognitive function. (A) Representative images of CBF changes before and after 2VO. (B) Changes in CBF in sham and 2VO rats. (C) Average escape latency for each group. (D) Representative swimming path of each group. (E) Time spent in target quadrant for each group. (F) Number of times crossing the platform for each group. (G) Average swimming speed for each group. *N* = 7. Data represent the mean ± SEM. n.s., nonsignificant; *p < 0.05; ***p < 0.001. CBF, cerebral blood flow; 2VO, permanent bilateral occlusion of the common carotid arteries; CCH, chronic cerebral hypoperfusion

To investigate whether bilateral occlusion of the common carotid arteries induced cognitive impairment in rats, we evaluated CBF using a laser Doppler flowmeter. Blood flow decreased from 133.00 ± 8.06 PU to 47.80 ± 6.51 PU after CCH for 2 h (Figure 1A). Furthermore, CBF decreased by more than 60% in the 2VO group (Figure 1B).

In the MWM test, on day 5 of the training phase, the 2VO group had a significantly longer escape latency to the platform compared with that of the sham group (Figure 1C). Representative images of traces in the spatial probe test are shown in Figure 1D. Compared with the sham group, the time spent in target quadrant (Figure 1E) and the number of times crossing the target (Figure 1F) were significantly lower in the 2VO group, which suggested that cognitive dysfunction was induced by CCH. Moreover, there was no significant difference in swimming speed between the two groups (Figure 1G), which indicated that MWM test performance was not influenced by differences in swimming, motor ability, or motivational deficits.

### 3.2 Cognitive recovery following administration of IMRCs

**FIGURE 2:**
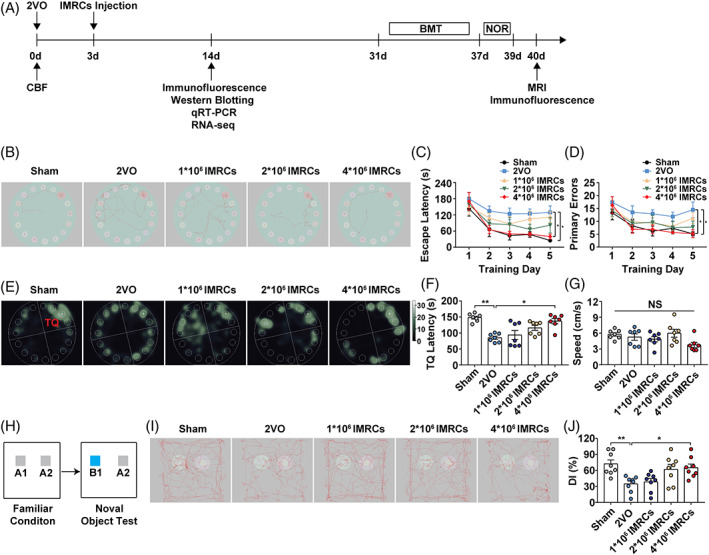
Cognitive recovery following administration of IMRCs. (A) The experimental schedule for injection of IMRCs and tests. (B–G) Results of the Barnes maze test among groups (*n* = 7). (B) Representative traces at day 5 of the acquisition phase. (C) Average escape latency for each group. (D) Average primary errors for each group. (E) Representative images of time spent in each of the four quadrants. (F) The amount of time spent in the TQ that initially contained the TH in the probe test phase. (G) Average swimming speed of each group. (H–J) Results of the NOR test among groups (*n* = 8). (H) Schematic of the NOR test. Grey square: familiar objects, blue square: novel objects. (I) Representative moving tracks in the NOR test. (J) The novel object discrimination index in each group. Data represent the mean ± SEM. n.s., nonsignificant; *p < 0.05; **p < 0.01. IMRCs, immunity‐ and matrix‐regulatory cells; TQ, target quadrant; TH, target hole; NOR, new object recognition

We examined whether injection of IMRCs could improve cognitive function of rats subjected to CCH. At day 3 post‐CCH induction, IMRCs were administered via tail‐vein injection at 3 doses. From day 14 to day 30, several tests were performed according to the experimental schedule (Figure 2A). We used the Barnes maze test (BMT) and the novel object recognition (NOR) test to evaluate cognitive function, as these tests are widely used to assess memory and spatial learning.^27^, ^28^, ^29^ During the first 4 days of the acquisition phase, there were no significant changes in primary escape latency or number of primary errors among these groups. As shown in Figure 2B, on training day 5, latencies and number of primary errors were significantly increased in 2VO group compared with those in the sham group, and administration of 4 × 10^6^ IMRCs resulted in significant decreases the time to enter the escape box (Figure 2C) and number of errors made (Figure 2D). In the subsequent probe test, the 4 × 10^6^ IMRCs group spent more time in the target quadrant than did the 2VO group (Figure 2F). Moreover, no significant differences were detected in the swimming speeds among the three groups (Figure 2G), which suggested that motor ability had no influence on BMT performance. Representative images of time spent in the target quadrant are shown in Figure 2E. In the NOR test, as shown in Figure 2I,J, rats treated with 4 × 10^6^ IMRCs exhibited significantly increased DI compared with that of the 2VO rats. These findings indicated that rats administrated 4 × 10^6^ IMRCs showed better cognitive abilities compared with the 2VO rats. Therefore, 4 × 10^6^ IMRCs/rat was used for the following experiments.

### 3.3 Immunity‐ and matrix‐regulatory cells alleviated myelin damage

**FIGURE 3:**
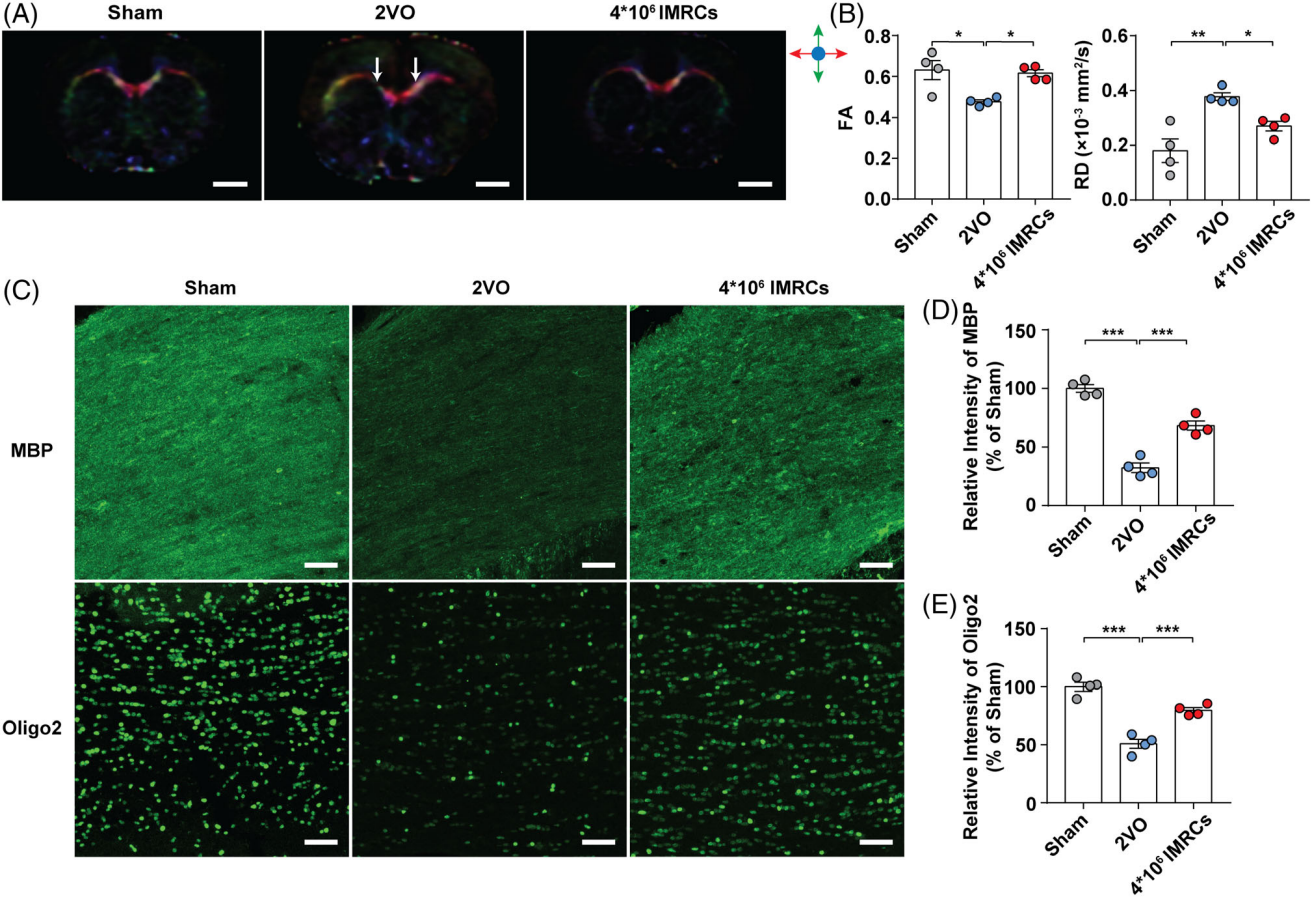
Immunity‐ and matrix‐regulatory cells alleviated myelin damage at 40 days after CCH. (A) Representative direction‐encoded FA colour maps in the corpus collosum. The directions of fibre tracks were colour‐coded with red for left–right, blue for anterior–posterior, and green for superior–inferior. White arrows indicated the injury sites. Scale bar = 20 mm. (B) FA and RD values in the corpus callosum of each group. (C) Representative images of MBP and Oligo‐ 2 staining in the corpus callosum. Scale bar = 50 μm. (D) The fluorescence intensity of MBP in the corpus callosum of each group, calculated as fold change compared to sham. (E) The fluorescence intensity of Oligo‐2 in the corpus callosum of each group, calculated as fold change compared sham. *N* = 4. Data represent the mean ± SEM. *p < 0.05; **p < 0.01; ***p < 0.001. FA, fractional anisotropy; RD, radial diffusivity; MBP, myelin basic protein; Oligo‐2, Oligodendrocyte lineage‐specific basic helix–loop–helix transcription factors

Given the relationship between white‐matter integrity and cognitive function,^8^, ^30^ we used DTI to evaluate the white matter integrity of the corpus callosum, which contains the most white matter tracts in the rat brain. Representative images are shown in Figure 3A. We observed that fractional anisotropy was significantly reduced and radial diffusivity was significantly elevated in the corpus callosum of 2VO rats 40 days after surgery compared with those in the sham group, which indicated that CCH induced white matter injury, especially in myelin sheaths (white arrows indicate the injury sites). Treatment with IMRCs reversed the white matter injury observed at 40 days post‐surgery (Figure 3B). We then evaluated myelin sheath loss in the corpus callosum and found that the intensities of MBP (Figure 3D) and Oligo‐2 (Figure 3E) were significantly decreased 40 days after CCH. Treatment with IMRCs significantly alleviated myelin damage, which suggested that IMRCs could prevent myelin damage 40 days after surgery. Representative images are shown in Figure 3C.

A previous study showed that the number of IMRCs decreased rapidly within 10 days after intravenous injection and remained low thereafter.^21^ Therefore, the effects of IMRCs on myelin loss was observed in the turning point. The results of MBP (Figure S1B) and Oligo‐2 (Figure S1C) staining showed a significant protective effect of IMRCs against myelin loss. The protein levels of MBP (Figure S1E) and Oligo‐2 (Figure S1F) determined using western blot were consistent with the staining results. These findings suggested that IMRCs protected against myelin damage 14 days after CCH.

### 3.4 Immunity‐ and matrix‐regulatory cells suppressed the activation of microglia in the corpus callosum

**FIGURE 4:**
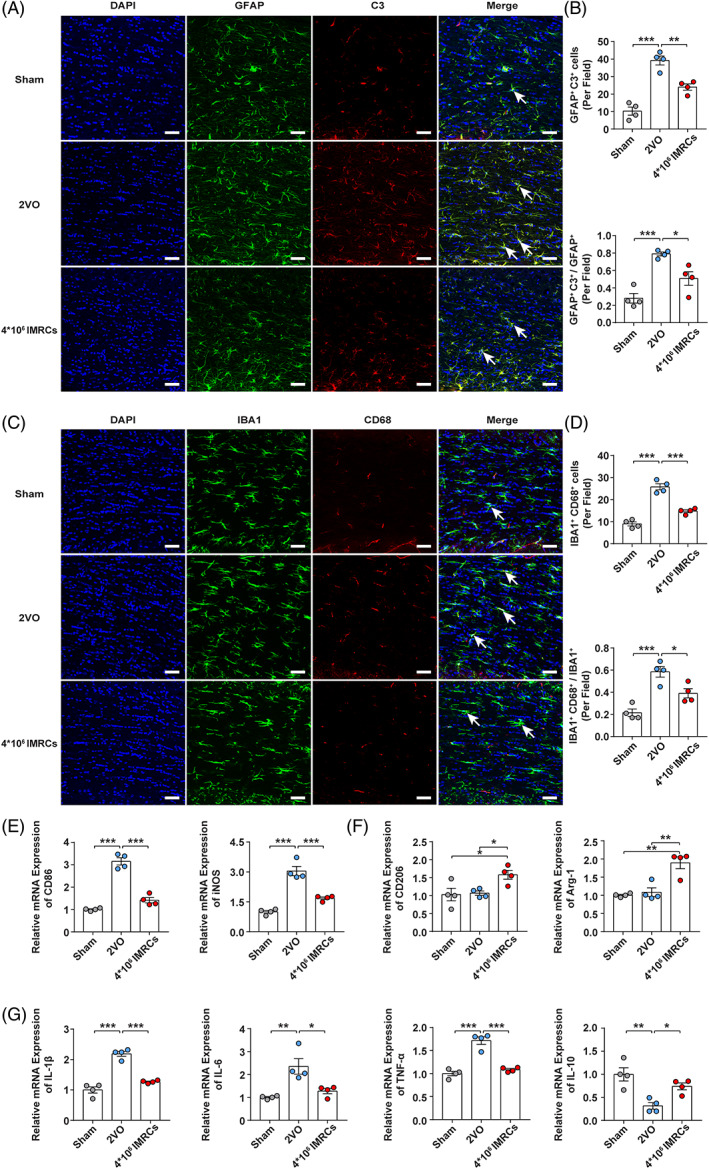
Immunity‐ and matrix‐regulatory cells suppressed activation of microglia in the corpus callosum at 40 days after CCH. (A) Representative images of DAPI (blue), GFAP (green), and C3 (red) immunofluorescence triple‐staining. (B) Quantification and proportion of GFAP^+^ C3^+^ A1 astrocytes in the corpus callosum of each group. (C) Representative images of DAPI (blue), IBA1 (green), and CD68 (red) immunofluorescence triple‐staining. (D) Quantification and proportion of IBA1^+^ CD68^+^ activated microglia in the corpus callosum of each group. (E) The mRNA levels of M1 (CD86, iNOS) and (F) M2 (CD206, Arg1) microglia markers in the corpus callosum of each group. (G) The mRNA levels of proinflammatory (IL‐1β, IL‐6, TNF‐α) and anti‐inflammatory (IL‐10) cytokines in the corpus callosum of each group. *N* = 4. Scale bar = 50 μm. Data represent the mean ± SEM. *p < 0.05; **p < 0.01; ***p < 0.001

A number of studies have shown that neuroinflammation could result in white matter injury.^31^ In the brain, astrocytes and microglia are the major contributors to the inflammatory response, and also play important roles in the progression of VCI.^8^ To investigate changes in neuroinflammation following administration of IMSCs, we examined activation of microglia and astrocytes in brain sections. Immunofluorescence staining showed that abundance of complement component 3 (C3)^+^ reactive astrocytes in the corpus callosum was significantly decreased in the IMRCs group compared with that in the 2VO group at days 14 (Figure S2A,B) and 40 (Figure 4A,B).

We further investigated the density and proportion of activated microglia by performing double immunostaining for CD68^+^ and IBA1^+^ cells, which are markers of active microglial phagocytosis.^32^ The proportion of CD68^+^/IBA1^+^ cells was significantly lower in the IMRCs group compared with that in the 2VO group at days 14 (Figure S2C,D) and 40 (Figure 4C,D). These findings suggested that administration of IMRCs ameliorated neuroinflammation in the CCH rat brain by regulating microglia and astrocyte.

Activated microglia exhibit the pro‐inflammatory M1 phenotype or the anti‐inflammatory M2 phenotype.^33^ We measured the mRNA levels of M1 markers (CD86, iNOS), M2 markers (CD206, Arg1), and inflammatory factors (TNF‐α, IL‐1β, IL‐6, IL‐10) in the corpus callosum using RT‐PCR at 14 days and 40 days after CCH. The results showed that, compared with the sham group, the levels of M1 markers (CD86, iNOS) (Figure 4E; Figure S2E) and pro‐inflammatory factors (TNF‐α, IL‐1β, IL‐6) (Figure 4G; Figure S2G) in the 2VO group were significantly increased. Moreover, anti‐inflammatory factors (IL‐10) (Figure 4G; Figure S2G) were significantly decreased in the 2VO group compared with those in the sham group. The results indicated that CCH induced transformation of microglia to the cytotoxic M1 phenotype, which was consistent with previous studies.^34^, ^35^ After injection of IMRCs, the levels of M1 microglia markers (CD86, iNOS) (Figure 4E; Figure S2E) and pro‐inflammatory factors (TNF‐α, IL‐1β, IL‐6) (Figure 4G; Figure S2G) were significantly decreased in the IMRCs group compared with those in the 2VO group. Furthermore, administration of IMRCs resulted in upregulation of the expression of M2 microglia markers (CD206, Arg1) (Figure 4F; Figure S2F) and anti‐inflammatory factors (IL‐10) (Figure 4G; Figure S2G) compared with those in the 2VO group. These results showed that IMRCs could suppress neuroinflammation and induce M1 to M2 microglial polarization following CCH. In addition, IMRCs were not detected by immunofluorescence after 30 days of intravenous injection in our study (Figure S3A,B), which is consistent with our previous conclusions.^21^

### 3.5 RNA‐seq analysis revealed transcriptional changes in the corpus callosum

**FIGURE 5:**
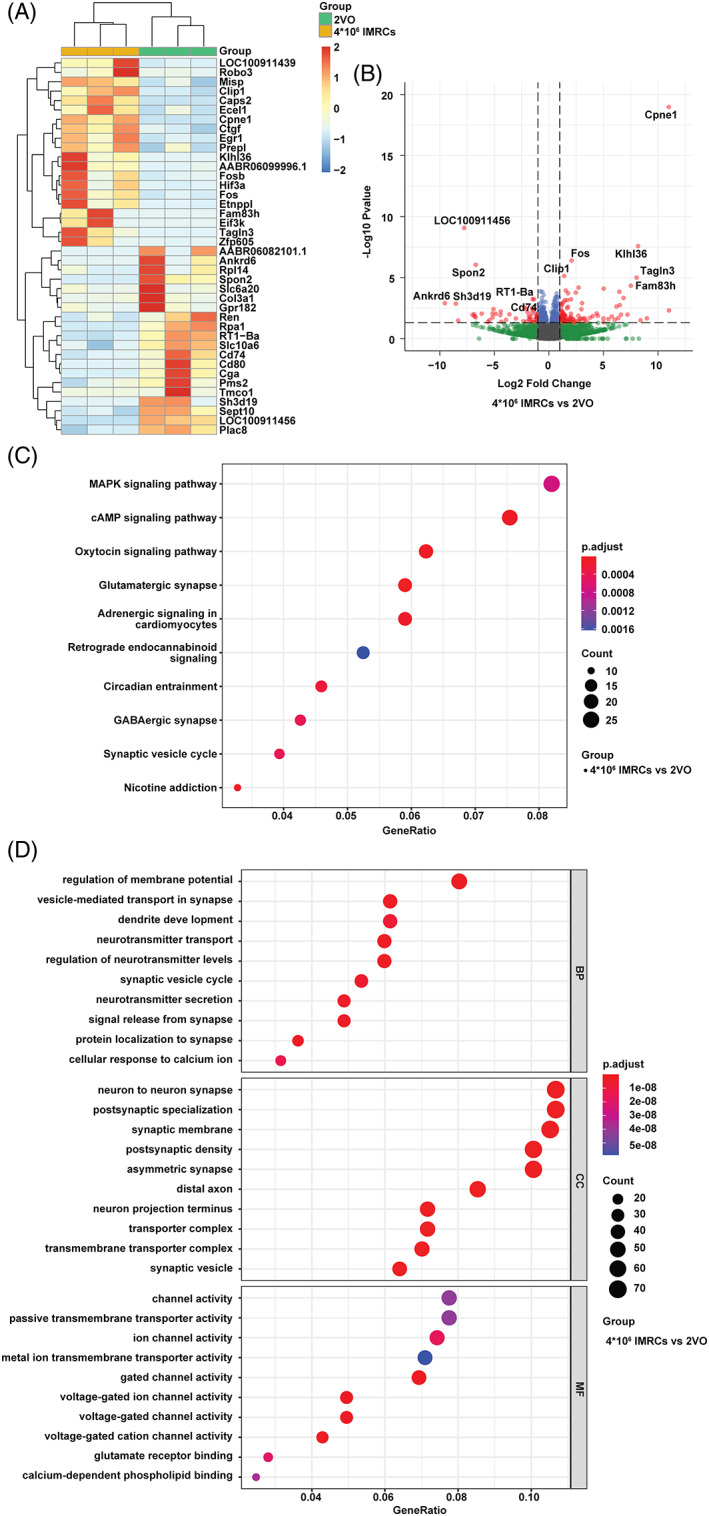
RNA‐seq of transcriptional changes in the corpus callosum. (A) Heatmap illustration showing differentially expressed genes (DEGs) between the IMRCs group and the 2VO group. Colouring indicates the log_2_‐transformed fold change. (B) Volcano plot showing major differentially expressed genes. (C) KEGG pathway analysis showed an enrichment of pathways involved in MAPK signalling. (D) GO pathway analysis of representative profiles of genes involved in biological process, cellular component, and molecular function. *N* = 3. BP, biological process; CC, cellular component; MF, molecular function

To further understand the role of IMRCs in CCH pathology, we performed RNA‐seq analysis of the corpus callosum in the 2VO (*n* = 3) and IMRCs (*n* = 3) groups. A heat map showed that six samples were clustered into two groups with distinct expression patterns, as determined by clustering analysis of DEGs (Figure 5A). After treatment with IMRCs, 107 genes were upregulated, and 54 genes were downregulated. We found that Cpne1 was the top‐ranked gene up‐regulated in the IMRCs group, which indicated that Cpne1 may be a potential target gene for VCI therapy (Figure 5B). We observed an enrichment of pathways involved in MAPK signalling (Figure 5C), a process that influenced neuroinflammation in the CCH model. The results from GO analysis included biological process (BP), cellular component (CC), and molecular function (MF) (Figure 5D).

## 4 DISCUSSION

Increasing evidence suggests that cerebral small vessel disease (SVD) is the primary cause of VCI.^36^ Chronic cerebral hypoperfusion models are widely accepted animal models of SVD^37^, ^38^ because sustained cerebral hypoperfusion leads to white matter attenuation, which has been shown to be a major feature common to SVD and Alzheimer's disease (AD).^39^ Moreover, CCH has also been considered to be a potential early contributor to AD.^40^ In our previous studies, IMRCs were used to treat AD mice via suppression of inflammation and activation of microglia.^21^, ^22^ In this study, intravenous injection of 4 × 10^6^ IMRCs/rat reversed CCH‐induced cognitive dysfunction. Our findings indicated that administration of IMRCs may be an effective cell‐based therapeutic strategy for treatment of VCI.

White matter integrity is critical for efficient neuronal communication and cognitive function.^41^ Damage to cerebral white matter, particularly myelin sheaths, is strongly associated with cognitive dysfunction and dementia.^42^, ^43^, ^44^ Clinical studies have demonstrated that white matter injury has been the most reliable etiological and prognostic indicator of cognitive disability.^45^, ^46^ Diffusion tensor imaging can be used to measure microstructural damage to white matter.^47^, ^48^ Fractional anisotropy and RD are two common indicators used to detect water molecule diffusion in brain tissue.^49^ In our study, reduced FA and increased RD in the corpus callosum of rats with CCH were indicative of white matter disintegration and demyelination. Furthermore, the loss of oligodendrocytes and attenuation of myelin density were observed in the earlier stages of CCH, which indicated that hypo‐myelination may precede cognitive impairments. These results were consistent with previous studies showing that damage to myelin was related to cognitive decline.^24^, ^50^ Treatment with IMRCs treatment reduced 2VO‐induced myelin damage, which indicated that IMRCs may protect against myelin damage.

Astrocytes and microglia are key inflammatory regulators in the central nervous system, and can be classified into neurotoxic (pro‐inflammatory) and neuroprotective (anti‐inflammatory) phenotypes.^51^ Activation of astrocytes and microglia is important in initiation and propagation of neuroinflammation in various neurological disorders.^52^, ^53^ In rats subjected to CCH, activated astrocytes and microglia were associated with impaired cognitive function and contributed to oligodendrocyte death.^54^, ^55^ Moreover, activation of astrocytes is typically related to activation of microglia.^56^ Inhibition of microglial activation has been shown to reduce damage to white matter and alleviate cognitive impairment.^57^ However, the effect of MSCs on activation and polarization of microglia in CCH models has not been characterized. Several reports have shown that primary MSCs exerted protective effects and improved learning and memory in rats subjected to CCH, but the mechanisms were not clear.^58^, ^59^ Histological analysis in this study indicated that intravenous injection of IMRCs inhibited microglial activation and promoted the transition of microglia polarization from the pro‐inflammatory to the anti‐inflammatory phenotype, which was consistent with our previous studies in an AD mouse model. Furthermore, the main advantage of using hESC‐derived MSC‐like cells is lower tissue‐related heterogeneity and the potential to achieve large‐scale production.^22^, ^60^

Copine 1 is an important Ca^2+^‐dependent, phospholipid‐binding protein present in a broad range of organisms, including mammals and humans.^61^ A previous study showed that CPNE1 repressed NF‐κB‐related transcription by inducing endoprotease processing of the p65 subunit within a conserved region.^12^ The NF‐κB signalling pathway plays an important role in CCH‐induced neuroinflammation, and may promote activation of astrocytes and microglia.^62^, ^63^ We observed significant upregulation of CPNE1 in the corpus callosum of rats subjected to CCH following injection of IMRCs, which indicated that CPNE1 may be a potential target for the therapeutic effects of IMRCs.

In conclusion, our findings demonstrated that intravenous injection of IMRCs attenuated white matter injury and improved cognitive function in rats subjected to CCH through inhibition of microglial activation and regulation of microglia polarization.

## CONFLICT OF INTEREST

The authors declare that they have no known competing financial interests or personal relationships that could have appeared to influence the work reported in this paper.

## AUTHOR CONTRIBUTIONS

YLW and YKW designed the study and revised the manuscript. FZ analysed the data and revised the manuscript for important intellectual content. YZ carried out experiments, collected the data, accomplished statistical analysis and drafted the manuscript. JW provided cell sources and contributed reagents/materials analysis tools. DL, JL, ZH, KL and XC collected and assembled the data. WC, LJ, LW, BH conceptualized and designed the study.

## Supporting information


**Figure S1** IMRCs alleviated myelin damage at 14 days after CCH. (A) Representative images of MBP and Oligo‐2 staining in the corpus callosum. (B) The fluorescence intensity of MBP in the corpus callosum of each group, calculated as fold change compared to sham. (C) The fluorescence intensity of Oligo‐2 in the corpus callosum of each group, calculated as fold change compared to sham. (D) Representative western blot of MBP and Oligo‐2 expression in the corpus callosum. (E) Semi‐quantitation of MBP in the corpus callosum of each group. (F) Semi‐quantitation of Oligo‐2 in the corpus callosum of each group. *N* = 4. Scale bar = 50 μm. Data represent the mean ± SEM. *p < 0.05; **p < 0.01; ***p < 0.001.

**Figure S2** Immunity‐ and matrix‐regulatory cells suppressed the activation of microglia in the corpus callosum at 14 days after CCH. (A) Representative images of DAPI (blue), GFAP (green), and C3 (red) immunofluorescence triple‐staining. (B) Quantification and proportion of GFAP^+^ C3^+^ A1 astrocytes in the corpus callosum of each group. (C) Representative images of DAPI (blue), IBA1 (green), and CD68 (red) immunofluorescence triple‐staining. (D) Quantification and proportion of IBA1^+^ CD68^+^ activated microglia in the corpus callosum of each group. (E) The mRNA levels of M1 (CD86, iNOS) and (F) M2 (CD206, Arg1) microglia markers in the corpus callosum of each group. (G) The mRNA levels of proinflammatory (IL‐1β, IL‐6, TNF‐α) and anti‐inflammatory (IL‐10) cytokines in the corpus callosum of each group. *N* = 4. Scale bar = 50 μm. Data represent the mean ± SEM. *p < 0.05; **p < 0.01; ***p < 0.001.

**Figure S3** Immunofluorescence analyses of immunity‐ and matrix‐regulatory cells in the corpus callosum at 30 days after CCH. (A) Representative images of DAPI (blue) and HNA (green) immunofluorescence double‐staining. (B) Representative images of DAPI (blue) and STEM121 (green) immunofluorescence double‐staining. *N* = 4. Scale bar = 50 μm. HNA, Human Nuclear Antigen; STEM121, a protein in cytoplasm of human cell.

## Data Availability

The datasets used or analysed in this study are available from the corresponding author on reasonable request.
