# Supplementary figures and images for: Human ESC‐derived immunity‐ and matrix‐ regulatory cells ameliorated white matter damage and vascular cognitive impairment in rats subjected to chronic cerebral hypoperfusion
Source: Cell Prolif. 2022 Apr 19;55(5):e13223. doi: 10.1111/cpr.13223 (PMC9136497; doi:10.1111/cpr.13223)

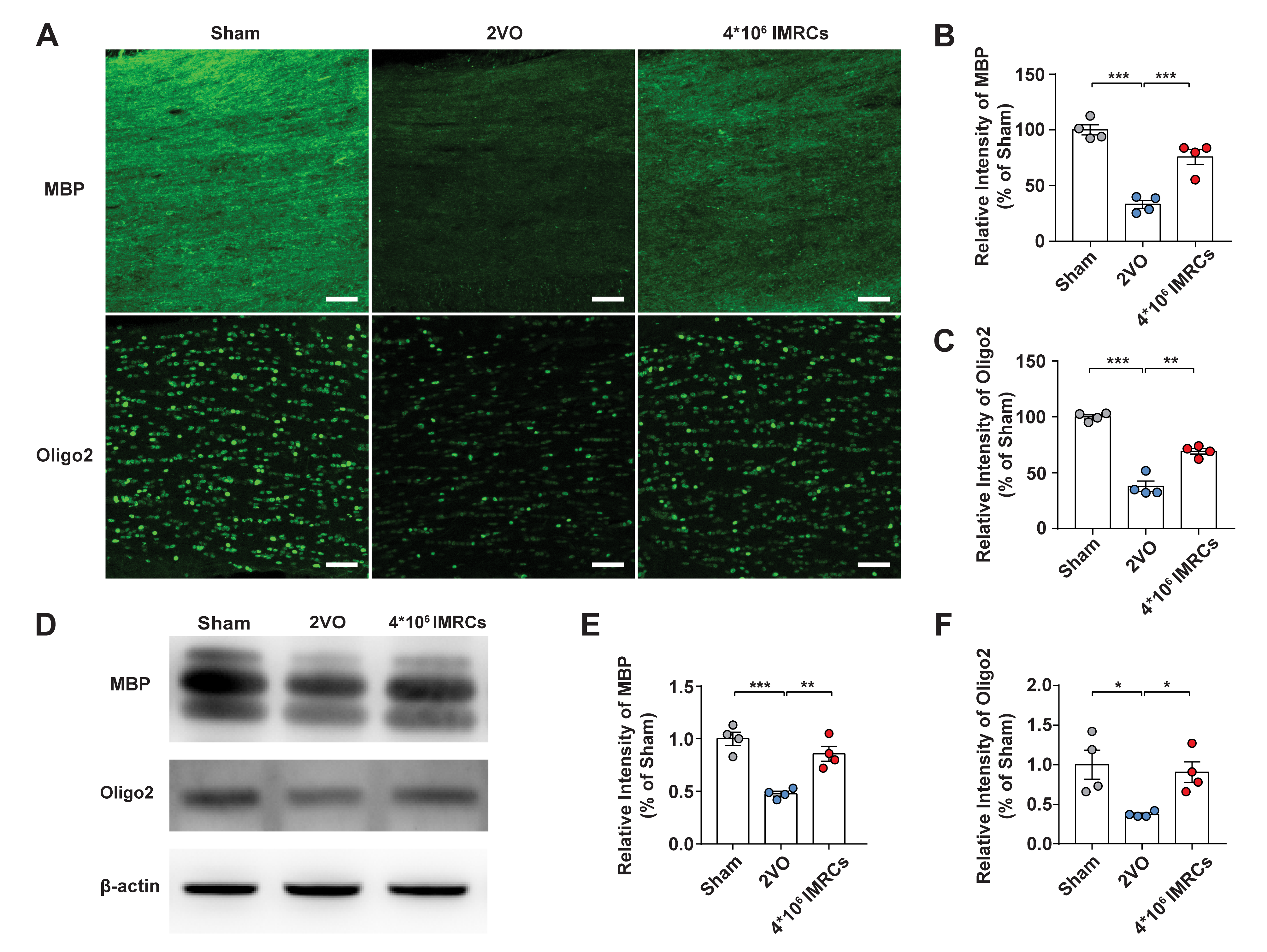

Supplement: Supplementary file 1 — Figure S1 IMRCs alleviated myelin damage at 14 days after CCH. (A) Representative images of MBP and Oligo‐2 staining in the corpus callosum. (B) The fluorescence intensity of MBP in the corpus callosum of each group, calculated as fold change compared to sham. (C) The fluorescence intensity of Oligo‐2 in the corpus callosum of each group, calculated as fold change compared to sham. (D) Representative western blot of MBP and Oligo‐2 expression in the corpus callosum. (E) Semi‐quantitation of MBP in the corpus callosum of each group. (F) Semi‐quantitation of Oligo‐2 in the corpus callosum of each group. N = 4. Scale bar = 50 μm. Data represent the mean ± SEM. *p < 0.05; **p < 0.01; ***p < 0.001. [file CPR-55-e13223-s002.tif]

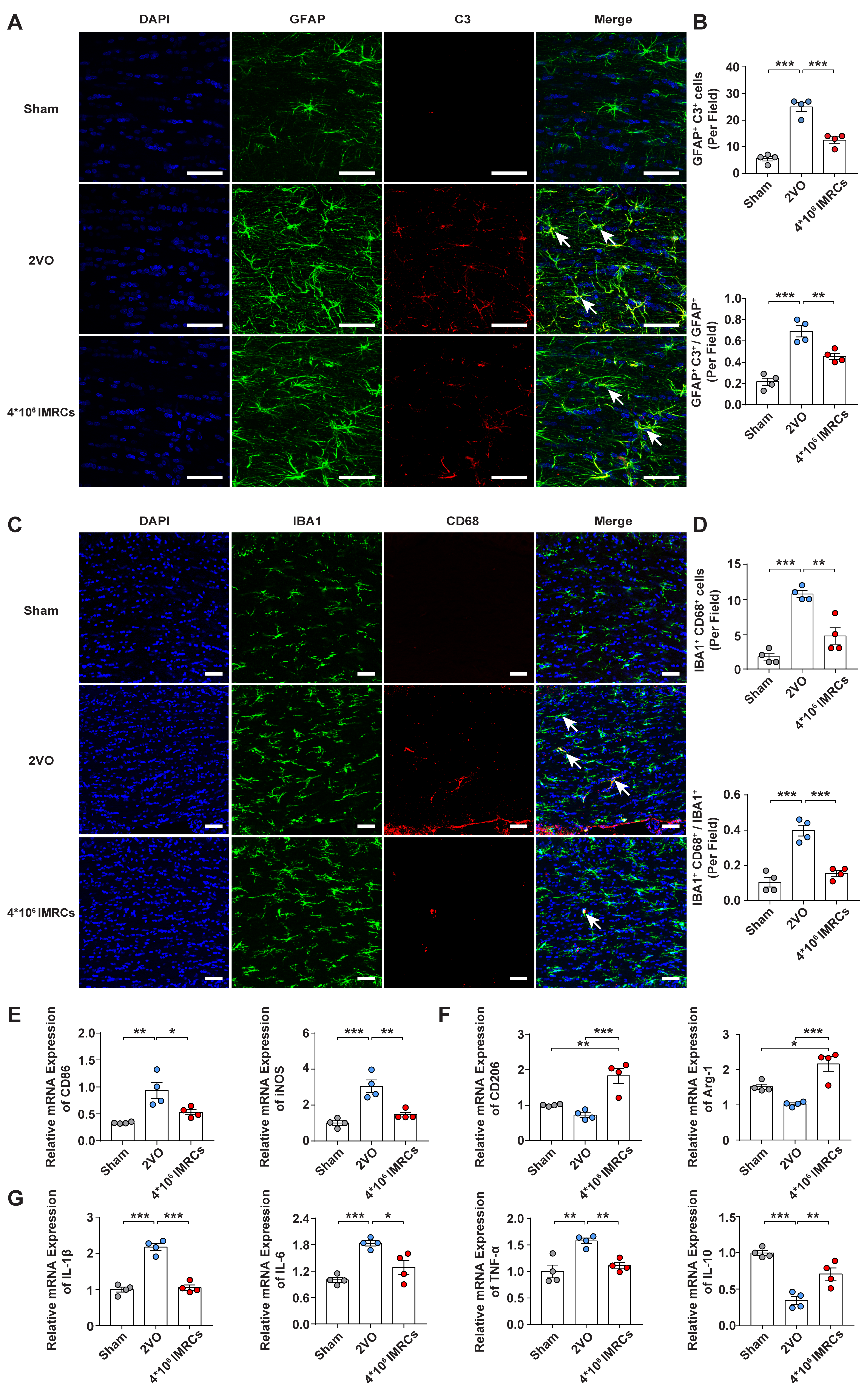

Supplement: Supplementary file 2 — Figure S2 Immunity‐ and matrix‐regulatory cells suppressed the activation of microglia in the corpus callosum at 14 days after CCH. (A) Representative images of DAPI (blue), GFAP (green), and C3 (red) immunofluorescence triple‐staining. (B) Quantification and proportion of GFAP+ C3+ A1 astrocytes in the corpus callosum of each group. (C) Representative images of DAPI (blue), IBA1 (green), and CD68 (red) immunofluorescence triple‐staining. (D) Quantification and proportion of IBA1+ CD68+ activated microglia in the corpus callosum of each group. (E) The mRNA levels of M1 (CD86, iNOS) and (F) M2 (CD206, Arg1) microglia markers in the corpus callosum of each group. (G) The mRNA levels of proinflammatory (IL‐1β, IL‐6, TNF‐α) and anti‐inflammatory (IL‐10) cytokines in the corpus callosum of each group. N = 4. Scale bar = 50 μm. Data represent the mean ± SEM. *p < 0.05; **p < 0.01; ***p < 0.001. [file CPR-55-e13223-s001.tif]

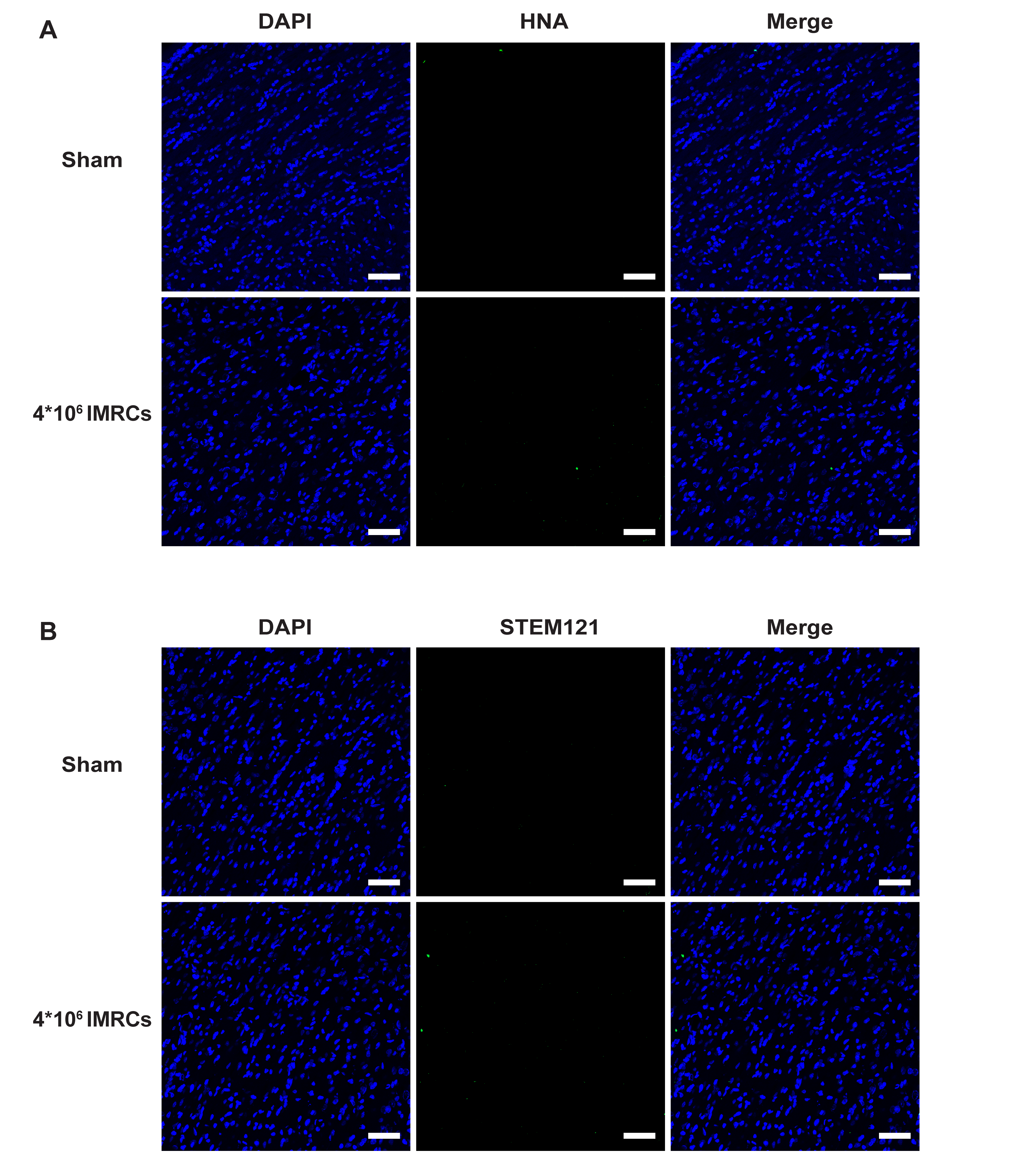

Supplement: Supplementary file 3 — Figure S3 Immunofluorescence analyses of immunity‐ and matrix‐regulatory cells in the corpus callosum at 30 days after CCH. (A) Representative images of DAPI (blue) and HNA (green) immunofluorescence double‐staining. (B) Representative images of DAPI (blue) and STEM121 (green) immunofluorescence double‐staining. N = 4. Scale bar = 50 μm. HNA, Human Nuclear Antigen; STEM121, a protein in cytoplasm of human cell. [file CPR-55-e13223-s003.tif]
